# Risk of Injury to Others: The Development of an Algorithm to Identify Children and Youth at High-Risk of Aggressive Behaviours

**DOI:** 10.3389/fpsyt.2021.750625

**Published:** 2022-01-03

**Authors:** Shannon L. Stewart, Angela Celebre, John P. Hirdes, Jeffrey W. Poss

**Affiliations:** ^1^Faculty of Education, Western University, London, ON, Canada; ^2^Faculty of Applied Health Sciences, University of Waterloo, Waterloo, ON, Canada

**Keywords:** children and youth, mental health, physical aggression, harm to others, interRAI

## Abstract

Youth violence is considered one of the most preventable causes of morbidity and premature mortality. Various risk factors have previously been identified, however, there is presently a crucial need to develop effective decision-support tools in order to identify children and youth at increased risk for violence. The current study utilised data collected from the interRAI Child and Youth Mental Health Screener (ChYMH-S), within the province of Ontario, to develop and validate a methodology for the purpose of identifying young persons who were at greater risk of harm to others. Additional data from 59 mental health agencies validated the algorithm, and it was found to be a strong predictor of harmful behaviour toward others. The RIO algorithm provides a valuable decision-support tool with strong psychometric properties that may be used to identify young persons who exhibit signs or symptoms associated with increased likelihood of harm toward others, in order to provide early intervention efforts for these vulnerable youth, thereby reducing the likelihood of future aggressive behaviours.

## Introduction

Childhood physical aggression is an important public health concern, as it has the potential to lead to more serious, violent behaviours, resulting in a plethora of adverse consequences (1, 2). Violence among youth is considered one of the most preventable causes of morbidity and premature mortality, with homicide continuing to be one of the leading causes of death for young people between the ages of 10–24 (3, 4). Notably, the World Health Organisation has made a dedicated effort to focus on delineating risk factors of youth violence over the recent decades, moving toward a prevention model for violence (5, 6). Through the identification of modifiable risk factors of youth violence, preventative strategies could be implemented to reduce risk of aggression toward others.

### Physical Aggression in Youth

While the age of onset for serious injury toward others typically does not occur before the age of 12, studies have found that the majority of children demonstrate physical aggression toward others by 17 months, although it is rare for such young children to cause serious harm (7–9). Most children will learn over time how to regulate their physical aggression; however, those who do not are at highest risk of engaging in serious violent behaviour later in life (2). From a developmental perspective, the precursors of chronic physical aggression are present before the child begins school, suggesting that the spontaneous onset of aggressive behaviours in school-aged children is quite atypical (2, 10, 11). Finally, as these precursors are present at such an early stage of life, it falls in line that a number of the most well-established risk factors can be found within the family context and environment [e.g., (12)].

### Potential Risk Factors Regarding Injury Toward Others

A number of family factors have been implicated in the development of physical aggression and subsequent violent behaviour in children and youth. Tremblay and colleagues (12) reported that at 5 months old, the best predictors of a high physical aggression trajectory were coercive parenting and family dysfunction. Moreover, children who have been physically abused within their home are more likely to exhibit aggressive behaviour at school, engage in serious violent acts during their teenage years, and commit violent crimes as adults (13–15). Studies have also found that poor family management practises, such as low parental supervision and monitoring, severe and inconsistent discipline, and unclear expectations predict delinquency later on (16, 17).

Certain individual characteristics and behaviours have also been linked to harm toward others. Poor impulse control and emotion regulation have been associated with violent behaviour from childhood through to early adulthood (18, 19). Research has also found that antisocial behaviour presenting early in life can predict future violence, with disruptive behaviour in childhood being one of the best predictors of violent offending during adolescence and adulthood, particularly for boys (20–22). Further, early-onset conduct problems, such as engaging in destructive behaviours, have repeatedly been identified as important predictors of future violent and criminal acts (23, 24).

Finally, the literature has found that injury toward others is an enduring and robust predictor of future violent acts (25). Children who exhibit chronic physical aggression during their elementary school years are more likely to continue engaging in physical violence during adolescence (2). Importantly, the link between early onset of aggression to more serious and chronic violent behaviour is evidenced in numerous studies; for example, the Denver Youth Study reported that 62% of children who engaged in violent behaviours at 9 years of age or younger became chronic violent offenders during adolescence (26–28). Another study reported that two-thirds of boys who were highly aggressive at 10–13 years of age had criminal records of violent offences by the age of 26. This represented a 6-fold increase compared to those who had low aggression (29). In addition to a history of aggressive behaviour, pro-violence attitudes are also linked to the onset and perpetration of violence among youth (23, 30).

The current study's objective was to develop and validate a methodology for identifying young persons who are at heightened risk of harming others utilising a comprehensive instrument used as standard of care in many mental health agencies within the province of Ontario, Canada. A validated methodological approach to identify adults who are at risk of harm to others (RHO) has previously been developed by interRAI (31). interRAI is an international non-profit collaborative committed to improving the lives of vulnerable persons across the lifespan. In particular, the interRAI child and youth suite of instruments was designed to facilitate a standardised, comprehensive, and coordinated approach to the delivery of mental health services for infants, toddlers, children and youth. An initiative was undertaken to develop a new decision-support algorithm for identifying youth at greatest risk of harm to others by harnessing the power of the existing interRAI assessment system, given that no system for such identification currently exists. A similar methodology utilised in the RHO was applied, creating the Risk of Injury to Others (RIO) algorithm, to assist service providers in determining whether a child or youth was at high risk of harming other individuals. This article describes the development and validation efforts of the RIO algorithm.

## Methods

### Sample

The method used in the development and validation of the RIO algorithm parallels that of an algorithm that we have previously developed, the Risk of Suicide and Self-Harm (RiSsK) (32). The following is an abbreviated version of the methodology. For a more detailed and comprehensive account of our Methods, please refer to our previous work (32).

The participants of this study were children and youth who received mental health services from Ontario health agencies. Notably, the study drew from four sample populations for the different stages of the methodology: (1) derivation, (2) validation, (3) descriptive analyses, and (4) longitudinal analyses. Data from the Child and Youth Mental Health Screener (ChYMH-S) (33) were used in both the derivation and validation stages. The RIO algorithm was derived using 60,414 records from 54,280 unique individuals, collected between September 1, 2015 and January 31, 2019. The participants had a mean age of 11.8 years with males comprising 49.8% of the sample (SD 3.74, range 4–18 years). Following the derivation stage, secondary data analyses were completed to validate the algorithm using 2,117 records from 2,098 unique individuals that were completed between February 1, 2019 and March 5, 2019. The participants had a mean age of 11.7 years with males comprising 49.0% of the sample (SD 3.67, range 4–18 years). Fifty-nine mental health organisations were included in the original development efforts of the RIO algorithm. There were no differences in the methods or sources between the derivation and validation samples.

In the post-scale development stage, two additional related sources of data were used to conduct (1) additional descriptive analyses related to diagnoses and (2) longitudinal analyses related to predictive validity. These data sources included the Child and Youth Mental Health (ChYMH) (34) and the Child and Youth Mental Health and Developmental Disability (ChYMH-DD) (35). To conduct the analyses related to diagnoses, a sample of 25,104 ChYMH and ChYMH-DD assessments on 13,899 unique individuals was used, completed between September 1, 2015 and January 31, 2019. The participants had a mean age of 12.1 years and males made up 57.0% of the sample (SD 3.51, range 4–18 years). To conduct the longitudinal analyses, a sample of 6,608 ChYMH-S, ChYMH, and ChYMH-DD assessments on 5,542 unique individuals was used, completed between November 4, 2015 and January 31, 2019. The participants had a mean age of 11.5 years and males made up 58.3% of the sample (SD 3.53, range 4–18 years).

The three aforementioned assessment tools (i.e., ChYMH, ChYMH-DD, and ChYMH-S) are used routinely as the standard of care in Ontarian mental health agencies. Thus, the inclusion criteria for this study consisted of children and adolescents between the ages of 4–18 years who presented at mental health facilities utilising the interRAI child/youth suite of instruments as standard of care.

### Measures

The ChYMH-S is a relatively new assessment instrument developed by interRAI, a non-profit collaborative that is composed of researchers and clinicians from over 35 countries. It is a brief assessment tool utilised to assess, triage, and prioritise children and adolescents seeking mental health services.

Nearly 100 items comprise the ChYMH-S. The items are generally selected from the larger comprehensive Child and Youth Mental Health assessment (34), with some additional items specific to screening purposes. The full interRAI ChYMH and ChYMH-DD assess mental health needs more extensively. These comprehensive tools consist of ~400 items that are used to assess psychiatric, social, environmental, and medical issues for children and youth. The ChYMH, ChYMH-DD, and ChYMH-S are divided into various subsections, such as mental state indicators, education, and behaviour. Further, the tool is supported by a detailed training manual containing coding rules for all items. The result is a reliable and valid assessment that can be used for a number of different purposes (e.g., case documentation and program planning) (36).

### Procedure

The ChYMH, ChYMH-S, and ChYMH-DD were routinely administered as part of the standard of care for young persons seeking mental health services in 59 agencies across the Province of Ontario. Assessors gathered information face-to-face or via telephone using a semi-structured interview format, from all available sources (e.g., conversations with parents/guardians, the child, and teachers; medical and education records; and clinical observations).

Secure web-based software was implemented to record assessment information. Before making the data available for analysis, personal identifiers were removed. Ethics approval was obtained from Western University's ethics review board to conduct secondary analyses on data collected in various Ontarian mental health agencies (REB #106415).

### Analysis

The intended use of the algorithm is to predict those at highest risk of injury to others based on an ordinal summary score in order to help facilitate early intervention efforts for these vulnerable youth. Assessors were asked to record perceived risk of “danger to others” using a single ordinal item that ranges in value from 0 (minimal) to 4 (very severe or imminent), based on all evidence available to the assessor at that time. We used this estimate as the dependent variable to be predicted by a variety of items from the ChYMH-S. As such, the dependent variable is a subjective professional opinion, as opposed to an objective behavioural measure of aggression. Because this scale is intended to be used with the comprehensive ChYMH assessment, all of the independent variables included in the algorithm must be available on both the ChYMH-S and full ChYMH. Notably, the single item for risk of danger to others is not recorded in the full ChYMH instrument, as it is in the ChYMH Screener. All of the screening records were used for scale development in order to properly represent the population of the sample. For example, if a young person has been screened twice, such as within an inpatient and outpatient setting, both of their records would be included.

While various modelling options were explored, it was ultimately decided to use the simple unweighted mean clinician rating of risk as a starting point in these analyses. Modelling was done using an interactive decision tree tool, which is supported by the SAS Enterprise Miner package (37). The software employs both Chi-Square Automated Interaction Detection (CHAID) and Classification and Regression Trees (CART) to create decision trees for categorical or continuous dependent variables. While it is possible to use a fully automated process to generate decision trees, our approach was iterative in nature using both clinical judgement and statistical criteria to identify potential splitting rules in developing the final decision tree. SAS defaults to propose binary splits for suggested independent variables, but we consistently checked to determine whether trichotomous or more granular splits were warranted. Enterprise Miner identifies candidate variables for splits in decision trees based on statistical criteria such as variance reduction (for continuous variables), Gini Impurity (for nominal variables), or chi-squared tests of significance (for binary variables). The software orders candidate variables based on the strength of their statistical performance for each split, but it also allows the analyst to specify other splits based on substantive reasons. In some cases, the decision related to a specific split in the tree may be based on expected performance across multiple nodes rather than a single node. This allows the user to interactively control which variable is selected and explore alternative trees before proceeding.

A key strength of decision trees, as opposed to conventional regression models, is that it can naturally handle complex interactions that can identify important subgroups that would be difficult to identify with simple two-way multiplicative interaction terms. The end result after the analyst sequentially divides all cases into their respective nodes is a tree with mutually exclusive and exhaustive classifications. Attention was paid to not “overfit” the model with unreasonably small terminal nodes in the decision tree. In addition, in the derivation process, Enterprise Miner provides real time feedback on performance of each split in a virtual hold-back sample, which allows the analyst to avoid specifying splits that will be unstable across samples.

In decision tree modelling, the initial splits are particularly important. Forced splits were considered as initial splits, such as age and sex, in addition to top-ranking variables; however, the forced splits were not selected in our decision tree model because they failed to offer any additional explanatory power. Moreover, they resulted in some fragmentation and small cell sizes in some of the tree's branches. The final tree model was subsequently tested among both age and sex groups.

An important goal within the design of our RIO algorithm was for the final ordinal scale to have a compact range: 7 groups (labels of 0–6). Due to the large sample and numerous explanatory variables, decision trees could have 30 or more terminal nodes. As a result, some of the nodes needed to be combined after modelling, which was achieved using weighted k-means clustering. The end product was a parsimonious tree for which the final nodes could be logically assigned to one of the 7 groups.

Multinomial logistic regression was then employed using the seven groups of the algorithm to test model fit of the dependent variable, as well as provide the c-statistic [area under the receiver operator characteristic (ROC) curve] and odds ratios. This was repeated using the validation sample, which consisted of new screener assessments that had accrued since the derivation data work had begun—approximately a 5-week period. The next steps used a sample of 25,104 ChYMH and ChYMH-DD assessments, in which the RIO scale was calculated, and additional descriptive analyses related to diagnoses were conducted. Further, using a sample of 6,608 ChYMH-S, ChYMH, and ChYMH-DD assessments, the RIO scale was calculated, and longitudinal analyses related to predictive validity were conducted. All available initial screener assessments were included at time point 1. These were linked to the next assessment (either a screener, ChYMH, or ChYMH-DD) at time point 2, which was between 31 and 120 days in the future. The association between the subjective assessor rating of “danger to others” at baseline and five measures of violence at follow-up was examined. Additionally, the association between the RIO score at baseline and five measures of violence at follow-up was examined. Analyses were performed using SAS 9.4 and SAS Enterprise Miner 14.1.

## Results

**Figure 1:**
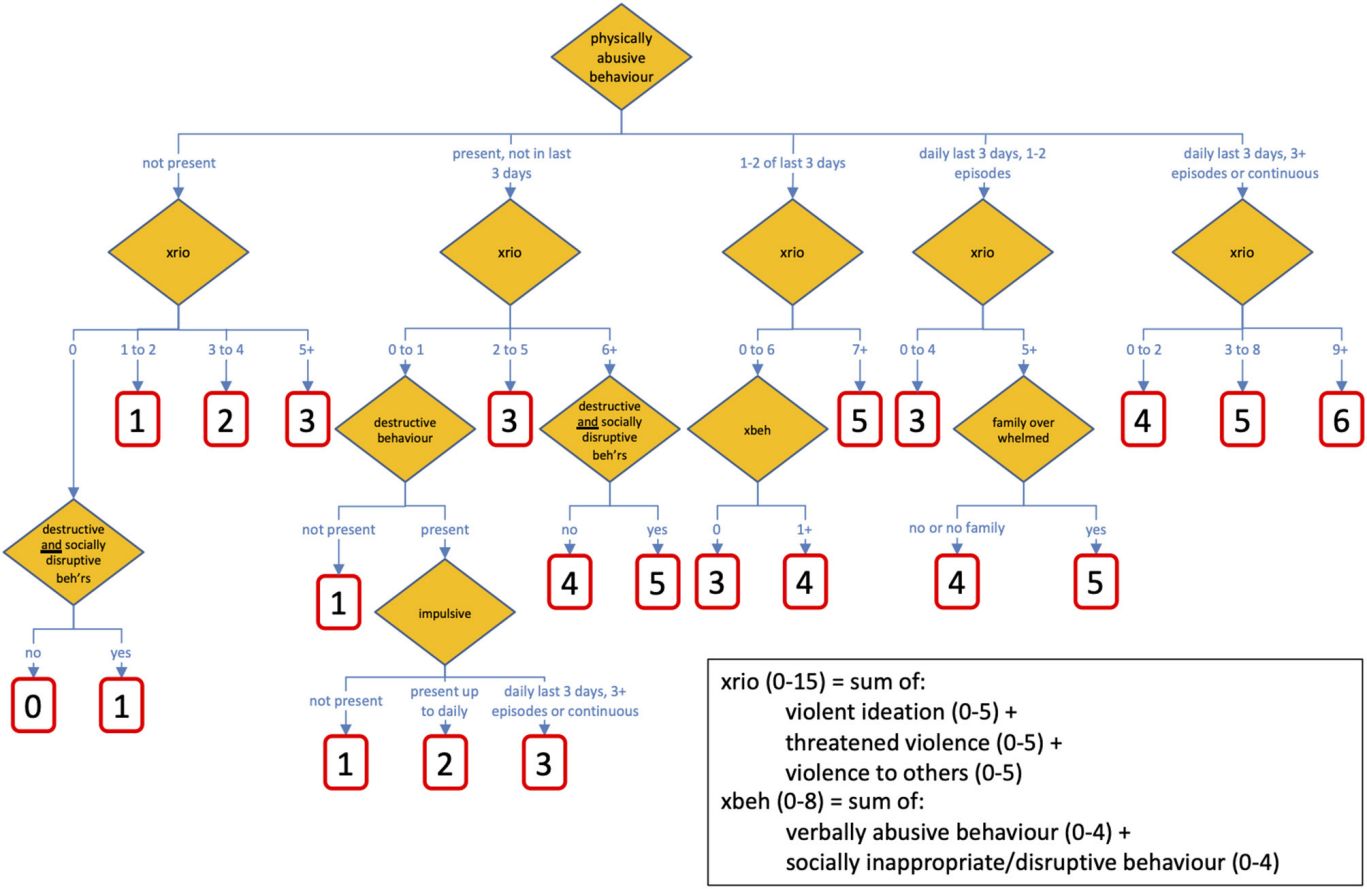
Risk of Injury to Others (RIO) decision tree diagram. *Socially disruptive beh'rs*, socially inappropriate/disruptive behaviours.

A schematic representation of the final RIO algorithm is presented in Figure 1. The RIO algorithm categorises young persons into levels of risk that suggest the need for heightened concern that the individual may be a danger to others, based on criteria as identified from the ChYMH-S. The final tree that was selected comprised of 21 terminal nodes, and used nine items from the ChYMH-S. All of the items included in the end product can be found on both the full ChYMH assessment and screener.

Groups were assigned a score from 0 (lowest) to 6 (highest) with higher scores indicating heightened risk of harm to others, as depicted in Table 1. The young person may fall into a given level via a number of different pathways that represent various combinations of the predictors. Highest risk was found in a small minority of young persons (~0.6% scored 6, the highest value on the RIO), in which 41.3% of these were rated as having a risk of harm to others that was severe, very severe, or imminent. Conversely, over half of those assessed were classified in the lowest risk group, in which only 0.08% were rated at these levels of risk. Table 1 shows the odds ratios of higher RIO levels, compared to the lowest group. The validation results for the 2,117 screening assessments are shown in Table 2. The C-statistic was 0.860 for the derivation sample and 0.853 for the validation sample.

**Table 1:** Derivation results of Risk of Injury to Others (RIO) algorithm (*N* = 60,414 screener assessments).

**Scale label**	**% of sample**	**Mean risk**	**% severe, very severe, or imminent risk**	**Odds ratio**	**Low 95% confidence interval**	**High 95% confidence interval**
0	54.5%	0.04	0.1%	Reference
1	12.2%	0.26	0.8%	8.3	7.6	9.0
2	5.7%	0.50	1.6%	18.4	16.8	20.2
3	16.6%	0.80	4.4%	36.4	33.8	39.2
4	5.3%	1.12	8.7%	69.3	63.3	75.9
5	5.1%	1.62	19.2%	171.6	156.2	188.5
6	0.6%	2.18	41.3%	477.4	392.0	581.4
				c-statistic = 0.860		

**Table 2:** Validation results of Risk of Injury to Others (RIO) algorithm (*N* = 2,117 screener assessments).

**Scale label**	**% of sample**	**Mean risk**	**% severe, very severe, or imminent risk**	**Odds ratio**	**Low 95% confidence interval**	**High 95% confidence interval**
0	56.5%	0.05	0.0%	Reference
1	11.9%	0.19	0.0%	5.0	3.2	7.8
2	6.3%	0.48	1.5%	13.4	8.4	21.2
3	12.6%	0.88	3.0%	36.7	25.2	53.5
4	8.3%	0.97	5.7%	43.7	29.0	65.8
5	3.8%	1.45	22.5%	117.0	69.9	195.6
6	0.6%	2.92	66.7%	>999.9	569.9	>999.9
				c-statistic = 0.853		

The derivation sample by age group and sex are presented in Tables 3 and 4, respectively. As shown in Table 3, younger children scored higher on the RIO algorithm than older children, indicating that they were judged to be at higher risk of harm to others. Specifically, for children 12 and older, only 3.2% were classified as a 5 or 6 on the RIO algorithm compared to children aged 8–11 years (8.1%) and those 7 and under (10.5%). As shown in Table 4, males scored higher on the RIO algorithm than females, with 8.4% of males classified as a 5 or 6 compared to only 3.2% of females.

**Table 3:** Risk of Injury to Others (RIO) algorithm by age, derivation sample (*N* = 60,414 screener assessments).

**Scale label**	**7 and younger**		**8–11**		**12 and older**	
	**% of sample**	**Odds ratio (95% CI)**	**% of sample**	**Odds ratio (95% CI)**	**% of sample**	**Odds ratio (95% CI)**
0	27.7%	Ref	42.7%	Ref	67.8%	Ref
1	14.6%	9.0 (7.01–11.50)	15.2%	8.8 (7.39–10.47)	10.1%	7.9 (7.05–8.82)
2	6.8%	20.7 (15.93–26.89)	6.4%	17.2 (14.16–20.81)	5.0%	18.7 (16.55–21.11)
3	30.0%	35.6 (28.44–44.68)	20.8%	38.0 (32.42–44.45)	10.7%	36.3 (32.92–39.98)
4	10.0%	58.9 (46.15–75.23)	6.9%	67.5 (56.33–80.83)	3.3%	84.7 (74.11–96.76)
5	9.5%	145.5 (113.52–186.55)	7.2%	158.2 (131.78–189.80)	2.9%	226.1 (195.72–261.19)
6	1.4%	407.5 (279.81–593.32)	0.9%	454.7 (325.50–635.26)	0.3%	619.5 (423.01–907.20)
c-statistic		0.792		0.841		0.867

**Table 4:** Risk of Injury to Others (RIO) algorithm by sex, derivation sample (*N* = 60,414 screener assessments).

**Scale label**	**Males**		**Females**	
	**% of sample**	**Odds ratio (95% CI)**	**% of sample**	**Odds ratio (95% CI)**
0	41.3%	Ref	67.5%	Ref
1	14.0%	6.7 (6.03–7.55)	10.4%	8.9 (7.81–10.14)
2	7.3%	14.4 (12.77–16.28)	4.1%	20.1 (17.33–23.39)
3	21.9%	29.3 (26.52–32.32)	11.4%	37.3 (33.31–41.75)
4	7.2%	52.4 (46.55–58.92)	3.6%	80.3 (69.48–92.84)
5	7.5%	131.8 (116.92–145.60)	2.8%	183.1 (156.25–214.53)
6	0.9%	342.0 (269.86–433.41)	0.4%	624.6 (433.82–899.31)
c-statistic		0.831		0.872

Further collapsing the RIO score into dichotomous groups, various cut-points of the scale were tested for their explanatory power of various levels of actual risk of injury toward others. Such cut-points would be employed to identify cases for specific services or referral related to harm to others. These results are summarised in Table 5. For flagging mild or moderate risk, a RIO cut-point of 2 or greater may be optimal, while for flagging severe risk, a RIO score of 3 or greater was found to perform best.

**Table 5:** Sensitivity and specificity results for the derivation sample: mild, moderate, and severe.

	**RIO**	**Sensitivity**	**Specificity**	**AUC**
Predict **mild or greater** risk of harm to others	1+	93.0%	68.9%	0.809
	2+	82.2%	81.5%	0.818
	3+	73.3%	86.2%	0.797
	4+	39.6%	94.4%	0.663
Predict **moderate or greater** risk of harm to others	1+	98.1%	60.6%	0.793
	2+	92.5%	73.5%	0.830
	3+	86.2%	79.1%	0.826
	4+	51.9%	91.0%	0.717
Predict **severe or greater** risk of harm to others	1+	98.4%	55.9%	0.772
	2+	94.7%	68.3%	0.815
	3+	91.4%	74.1%	0.827
	4+	63.4%	87.9%	0.773

*AUC, area under the curve*.

Using the ChYMH and ChYMH-DD assessment data, which include the nine items necessary to assign the RIO scale, diagnoses associated with higher RIO scores were investigated. As can be seen from Table 6, the most prevalent diagnoses associated with higher risk of harm to others were Disruptive Behaviour, Reactive Attachment, Substance-Related, and Attention Deficit/Hyperactivity disorders. Diagnoses associated with lower RIO scores were Eating, Mood, and Anxiety disorders.

**Table 6:** Risk of Injury to Others (RIO) algorithm by DSM diagnosis.

**DSM-IV^a^ N=25,104 full ChYMH or ChYMH-DD**	**RIO 2+**		**RIO 3+**	
	**Most important dx**	**Any importance**	**Most important dx**	**Any importance**
Disruptive behaviour	73.9%	67.2%	66.2%	58.6%
Reactive attachment	63.0%	65.9%	55.7%	58.1%
Substance related	64.0%	61.5%	55.7%	51.3%
Attention deficit hyperactivity	56.0%	55.6%	47.7%	47.4%
Autism spectrum	56.7%	54.7%	48.4%	46.5%
Learning or communication	50.8%	51.3%	43.8%	44.4%
Adjustment	40.3%	44.4%	33.5%	37.2%
Sleep	44.9%	39.0%	39.1%	31.7%
Schizophrenia/psychotic	41.9%	40.3%	32.3%	33.3%
Anxiety	31.4%	36.6%	26.2%	30.5%
Mood	30.5%	32.9%	24.8%	26.7%
Eating	12.3%	21.9%	11.3%	17.7%

a *Among assessments with this diagnosis, this is the proportion reaching this RIO threshold*.

**Figure 2:**
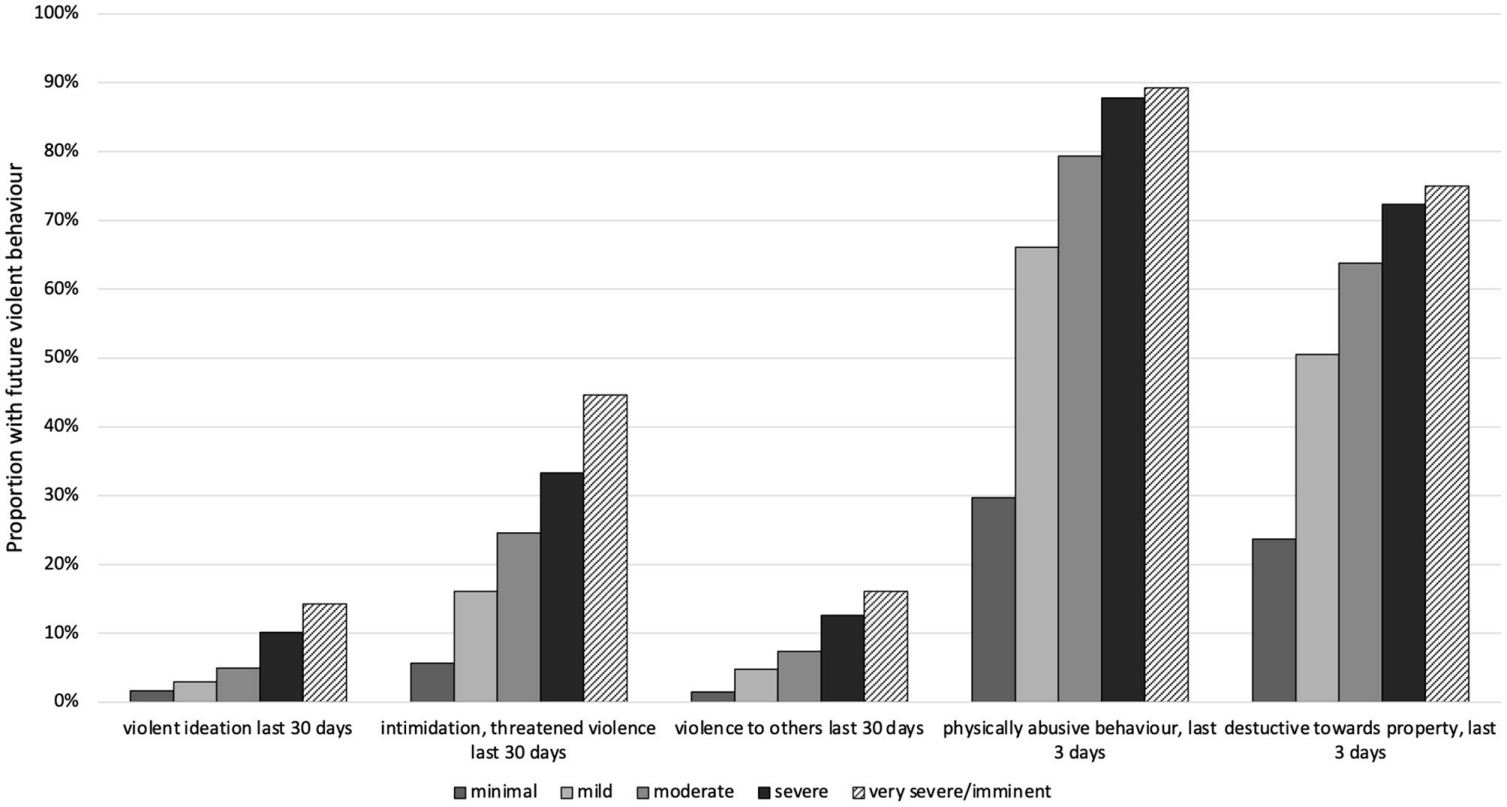
Longitudinal analysis: High risk for future violent behaviour by subjective assessor rating of “danger to others” at baseline (*N* = 6,608).

**Figure 3:**
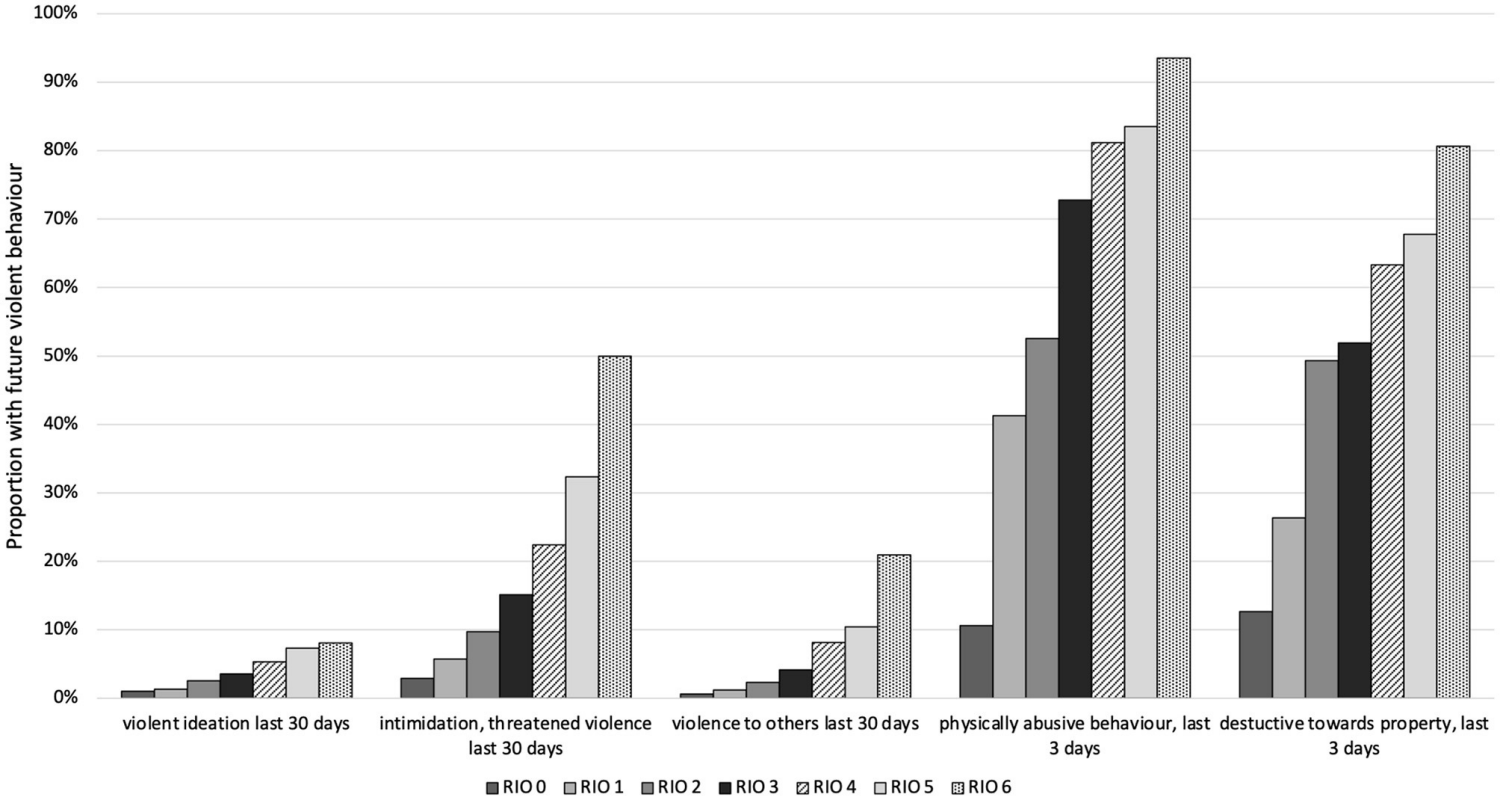
Longitudinal analysis: High risk for future violent behaviour by Risk of Injury to Others (RIO) score at baseline (*N* = 6,608).

Using the ChYMH, ChYMH-S, and ChYMH-DD assessment data, the predictive validity of the subjective assessor rating of “danger to others” and the RIO score were investigated. As can be seen from Figure 2, the “danger to others” rating is predictive of 5 future violent behaviour items (C-statistics of 0.66–0.72). Further, as shown in Figure 3, RIO itself is highly predictive of these 5 behaviour items (C-statistics of 0.70–0.83). Of note is that 8 of these 10 C-statistics are 0.7 or greater, suggesting a good model (38).

## Discussion

A variety of factors predicted high risk of injury to others. Several of the contributors were related to a prior history of abusive behaviours and violent thoughts/actions, certain individual traits and behaviours, and family factors. Young persons who displayed violent ideation, threatened violence, or engaged in violent actions toward others received higher scores on the RIO algorithm. This strong relationship between prior ideas, threats, and acts of violence, and future risk of injury toward others is well-supported by the literature. In a comprehensive multivariate analysis, one of the most salient predictors of harm toward others was prior violent behaviour, among both boys and girls (39). Research has also shown that the frequency of violent threats is positively associated with engagement in violent acts (40). Finally, in a comprehensive review of the literature, Hawkins and colleagues (41) reported that youth who have favourable attitudes toward violence were more likely to commit violent acts in the future.

We found that children and youth who exhibited physically abusive behaviour were at higher risk of injury toward others, and this was, in fact, the first predictor included within the RIO algorithm. This is in line with prior research that has found physical aggression to be the most consistent predictor of future violent offending (2, 42). In addition to physical abuse, verbally abusive behaviour was also associated with increased risk of harm toward others, in our model. This is consistent with extant literature that reported adolescents in the sixth grade who engaged in bullying, which encompassed both physical and verbal abuse (e.g., picking on another kid, slapping, or hitting), were more likely to be perpetrators of dating violence by the eighth grade (43). Children and youth who were more impulsive also received higher scores on the RIO algorithm, which is similar to previous work that has found a strong correlation between impulsivity and aggressive behaviours toward others (41, 44).

Socially inappropriate/disruptive behaviours, as well as destructive behaviours, also significantly contributed to higher scores on the RIO. This finding is in accordance with prior research, which has reported that a range of anti-social behaviours, including under-age smoking, stealing, and destruction of property, are linked to greater risk of violence among males (45, 46). Studies have also found that deficits in social and cognitive ability in childhood are associated with future aggressive behaviour (47, 48). Children who struggle with social and cognitive functioning may not be able to fully comprehend social norms, thereby acting in socially inappropriate and disruptive ways. Interestingly, belief and commitment to a social/moral order is suggested to decrease risk of engaging in violent behaviour (49). Therefore, children and youth who act in ways that would be viewed as contradictory to social norms may be at increased risk of harm toward others, which would support the current study's finding.

The last predictor variable of the RIO algorithm is the family being overwhelmed by the child or youth's condition, which could be due to a number of different reasons, including ineffective coping strategies when dealing with the young person's difficult presentation. Other family stressors may increase the distress level in an expediential manner, further taxing the situation. Research has shown that a number of family factors can increase a child's risk of engaging in injury toward others, such as low parental supervision and monitoring (17). Interestingly, a chaotic family life has been shown to increase risk of youth violence (50). Families may feel stressed because of the chaotic nature of their family environment, thus contributing to the child's increased risk of injuring others, a finding consistent with this study. Research has also found that harsh and inconsistent discipline is associated with aggression in children [e.g., (51)]. It can be postulated that when a family is overwhelmed by the child's situation, caregivers may not feel capable of providing the fair, consistent discipline the child requires for positive development, thereby resulting in a higher likelihood of the young person engaging in harmful behaviours.

Findings also indicated an association between age and scores on the RIO algorithm, whereby younger children were more likely to have a higher RIO score compared to older children. More specifically, younger children were at heightened risk of engaging in behaviours that would injure others compared to their older counterparts, a finding that is consistent with extant literature (52). However, it is important to note that although younger children tend to be more physically aggressive, older children and youth are larger in stature and size; therefore, their aggressive behaviours could lead to more serious injuries in others, despite the fact that it occurs less frequently. This relationship between age and severity of aggressive behaviour is well-documented in the literature, with the period of adolescence and young adulthood being known as a time of heightened risk behaviour, such as engaging in more violent acts. It has been reported that the age of onset for serious, violent offending typically does not occur before the age of 12, but this rate increases drastically from 12 to 16 years of age, doubling between 13 and 14 years old (7, 53). Therefore, it is critical to make the distinction that, although younger children are more likely to be physically aggressive, older children are more likely to engage in more serious, violent acts.

The current study also examined DSM-diagnoses related to the RIO algorithm, and identified disruptive behaviour, reactive attachment, substance-related, and attention deficit hyperactivity among the top diagnoses associated with higher RIO scores. The association between these diagnoses and higher risk of harm to others is well-supported by the literature. For example, one study that examined the most common psychiatric disorders among children and adolescents referred to mental health services for serious aggressive behaviour found that the most common diagnoses behind aggression were oppositional defiant disorder (93.02%), attention deficit hyperactivity disorder (88.37%) and conduct disorder (38.75%) (54). It has also been reported that aggression is a frequently co-occurring condition to reactive attachment disorder (RAD) (55). Several other studies have consistently found aggression to be one of the key risk factors associated with substance-use disorder (56, 57); furthermore, aggression has been found to be significantly related to early substance use initiation among youth (58).

Finally, the current study also investigated the predictive validity of the subjective assessor rating of “danger to others” as well as the RIO score itself. First, the findings showed that the assessors' rating of perceived risk of “danger to others,” subjective as it is, predicts future violent behaviour. More specifically, higher assessor ratings at time point 1 were associated with increased violent ideation, intimidation/threatened violence, violence to others, physically abusive behaviour, and destructive behaviour toward property at time point 2. This supports the use of this measure in the derivation of RIO. It may not be a “gold standard,” but it supports the validity of a measurement that predicts what is intended to be modelled. Second, the findings indicate that the RIO score itself at baseline is associated with increased violence in the future across the same five items previously described. This demonstrates the utility of the RIO algorithm in predicting future aggressive behaviour toward others among children and youth.

### Use and Utility of RIO

Based on our results, RIO is an empirically based decision-support tool that may be used to identify young persons who have a higher likelihood of engaging in harmful behaviour toward others. Because it can validly and reliably predict high-risk physically aggressive behaviour, mental health professionals will be able to make more systematic evaluations in determining whether an individual is at heightened risk of committing violent or injurious acts. Ultimately, the algorithm was designed to help facilitate early intervention efforts to provide support for these vulnerable youth in order to decrease the likelihood of future aggression.

Importantly, the use and utility of the RIO algorithm falls in line with that of our previously published RiSsK algorithm (32). For example, the RIO score can similarly be obtained automatically when the ChYMH-S assessment is submitted from the assessor's computer, and these results are also intended to be used along with other information obtained during the screening process.

Subsequent care planning steps are informed by whether the young person's score falls within the lower or upper range. If a score falls within the lower range, the clinical team should discuss further to decide whether, based on all available information, the RIO level seems appropriate. If a score falls within the upper range, the clinical team should consider the individual to be at high risk of injuring others. Clinicians can use the Harm to Others collaborative action plan (CAP) developed by interRAI to assist with their care planning (59, 60). When the young person is at high risk of injuring others, immediate intervention for acute physical aggression is required, followed by debriefing discussions and assessment of the incident. Regardless of moderate or high risk, it is imperative the clinician performs an assessment of harmful behaviour (e.g., precipitating factors, targets, intensity, frequency, and duration of episodes); this information will be used in the selection of subsequent interventions.

Similar to the RiSsK algorithm, the RIO also has broader applications beyond individualised care planning. For example, it can provide high-quality standardised data across large catchment areas, which would enable the identification of risk of injury to others across the system (e.g., examining different jurisdictional patterns); it can also be used to provide justification for specific services and expenditures, as well as for benchmarking purposes (61, 62). For a more detailed discussion of these broader applications, please refer to our previous work (32).

The major advantage of implementing the RIO algorithm would be that young persons with higher levels of risk should be receiving more emergent services and extensive resources (e.g., inpatient services) than those with lower-level risk. Nevertheless, this does not prohibit the likelihood of receiving appropriate services for young persons scoring at the lowest level of risk. Notably, research has shown that early identification and intervention can lead to reduced likelihood of future aggresssive behaviours [e.g., (63, 64)].

While there are a number of strengths in the current study, including internationally-used comprehensive assessment tools and the relatively large sample size, it also has limitations. For example, because all of the children and adolescents assessed were accessing inpatient or outpatient mental health services (i.e., entering the formal system), the results may not be generalizable to a community-based, non-clinical sample. As such, future research could examine whether the present study's findings are consistent when the sample population is from the community.

Additionally, assessors completed the items used to derive the RIO algorithm at the same time the overall risk score was determined utilising the interRAI ChYMH Screener. The algorithm was modelled on this overall risk score and, as a result, utilised concurrent measurement. Notably, while it may not have the ideal characteristics of an independent gold-standard measure on which to derive the RIO score, the validation efforts also utilised other instruments within the suite (e.g., ChYMH, ChYMH-DD) that did not incorporate the overall risk score, providing additional evidence of its utility. This approach was viewed as reasonable given the goals of the algorithm and its use across numerous instruments within the interRAI suite of child and youth assessments. Further concurrent validity measures were also examined within the ChYMH and ChYMH-DD that were not in the interRAI ChYMH Screener at the time the overall risk index was obtained by assessors; this included the items known to use/carry weapons and serious injury to another in the last 90 days. Furthermore, the present study investigated concurrent validity cross-sectionally among first assessments of individuals as well as predictively (using RIO at baseline and its association with these two items at a follow-up assessment between 31 and 182 days). Findings indicated strong concurrent validity.

## Conclusion

The adverse consequences of injury toward others are wide-ranging, including psychopathology, substance use, reduced psychosocial functioning, and the most severe and tragic consequence being youth homicide (65–68). In light of the negative sequelae of youth violence, identifying risk factors associated with harmful behaviour is crucial for the development of strategic prevention and intervention programs. This underscores the critical utility of the RIO algorithm, as it provides a psychometrically sound decision-support tool that can assist clinicians in identifying children and adolescents at heightened risk of injuring others, thus facilitating earlier intervention.

## Data Availability Statement

The datasets presented in this article are not readily available because of privacy restrictions and regulations. Requests to access the datasets should be directed to the corresponding author.

## Ethics Statement

The studies involving human participants were reviewed and approved by Western University's Ethics Review Board (REB #106415).

## Author Contributions

SS contributed to the conceptual basis of the study and its methodology. SS and JP developed the analytical strategy. JP performed the statistical analysis. All authors contributed to the formulation of the ideas presented in the study, provided critical feedback to the manuscript, were involved in the writing, and reviewing of the final manuscript.

## Funding

Public Health Agency of Canada (#1617-HQ-000050).

## Conflict of Interest

The authors declare that the research was conducted in the absence of any commercial or financial relationships that could be construed as a potential conflict of interest.

## Publisher's Note

All claims expressed in this article are solely those of the authors and do not necessarily represent those of their affiliated organizations, or those of the publisher, the editors and the reviewers. Any product that may be evaluated in this article, or claim that may be made by its manufacturer, is not guaranteed or endorsed by the publisher.

## Appendix

**TABLE A1:** Items used in the RIO algorithm.

**Item**	**Coding**
• Physical abuse—e.g., others were hit, shoved, scratched, sexually abused • Verbal abuse—e.g., others were threatened, screamed at, cursed at • Socially inappropriate or disruptive behaviour—e.g., screamed out during class, smeared or threw food or feces • Destructive behaviour toward property—e.g., throwing or breaking objects, turning over beds or tables, vandalism • Impulsive—e.g., running into traffic; takes risky actions without thinking; difficulty taking turns; interrupts	0. Not present 1. Present but not exhibited in last 3 days 2. Exhibited on 1–2 of last 3 days 3. Exhibited daily in last 3 days, 1–2 episodes 4. Exhibited daily in last 3 days, 3 or more episodes or continuously
• Violent ideation—e.g., reports of premeditated thoughts, statements, plans to commit violence • Intimidation of others or threatened violence—intentionally makes threatening gestures, verbalizations or stance with no physical contact (e.g., throwing furniture, explicit threats of violence) • Violence to others—acts with purposeful, malicious, or vicious intent, resulting in physical harm to another (e.g., stabbing, choking, beating)	0. Never 1. More than 1 year ago 2. 31 days - 1 year ago 3. 8–30 days ago 4. 4–7 days ago 5. In last 3 days
• Family members report feeling overwhelmed by child's/youth's condition—e.g., severe behaviour problems	0. No 1. Yes 8. Not applicable
